# Autologous minced cartilage implantation for treatment of focal cartilage defects of the glenohumeral joint results in good clinical and radiological outcomes after a minimum 2‐year follow‐up

**DOI:** 10.1002/jeo2.70803

**Published:** 2026-06-15

**Authors:** Bastian Scheiderer, Thilo Demmer, Lucca Lacheta, Klaus Woertler, Sebastian Siebenlist, Lukas Nawid Muench

**Affiliations:** ^1^ Department of Sports Orthopaedics, TUM University Hospital Technical University of Munich Munich Germany; ^2^ Department of Orthopaedics and Trauma Surgery University of Cologne Cologne Germany; ^3^ Musculoskeletal Radiology Section, TUM University Hospital Technical University of Munich Munich Germany

**Keywords:** arthroscopy, cartilage defect, glenohumeral joint, minced cartilage implantation, shoulder

## Abstract

**Purpose:**

Autologous minced cartilage implantation (MCI) has been proposed as a promising single‐step procedure for treatment of focal cartilage defects in the knee joint. However, clinical evidence regarding the glenohumeral joint is limited. The purpose of the present study was to evaluate clinical and radiological outcomes after MCI for focal cartilage defects of the glenohumeral joint.

**Methods:**

Patients who underwent arthroscopic MCI for focal, grade IV cartilage defects of the glenohumeral joint between October 2021 and March 2023 were analysed. Postoperative clinical evaluation included the Constant–Murley (CM), American Shoulder and Elbow Surgeons (ASES) and Disability of the Arm, Shoulder and Hand (DASH) score, the Simple Shoulder Value (SSV) and assessment of range of motion (ROM) and strength. Postoperative shoulder‐dependent athletic ability was evaluated using the Athletic Shoulder Outcome Scoring System (ASOSS). Cartilage repair tissue morphology was assessed on magnetic resonance imaging using the Magnetic Resonance Observation of Cartilage Repair Tissue (MOCART) 2.0 score.

**Results:**

All eight eligible patients (median age at surgery, 25.5 ± 5.5 years) were included in the study, with a median follow‐up of 2.5 years (range, 2.0–2.8 years). The focal cartilage defect was located at the glenoid concavity in six patients and humeral head in two patients, with a median size of 2.4 cm^2^ (range, 1.0–4.0 cm^2^). At final follow‐up, patients showed a median CM score of 85.0, CM score relative to the contralateral side of 95.5, ASES score of 93.5, SSV of 90.0 and DASH of 4.6. Patients demonstrated no difference in ROM and strength compared to the contralateral side (*p* > 0.05, respectively). With a median ASOSS of 86.0, patients showed good athletic ability. The median MOCART score was 74.2 with substantial defect filling and integration to adjacent cartilage.

**Conclusion:**

MCI for treatment of focal cartilage defects of the glenohumeral joint showed a radiologically successful defect coverage along with good to excellent clinical outcomes in patients with or without concomitant procedures at a minimum 2‐year follow‐up.

**Level of Evidence:**

Level IV, retrospective case series.

**Abbreviations**
ACI: autologous chondrocyte implantation
ASES: American Shoulder and Elbow Surgeons
ASOSS: Athletic Shoulder Outcome Scoring System
CI: confidence interval
CM: Constant–Murley
DASH: Disability of the Arm, Shoulder and Hand
ICC: interclass correlation coefficient
LHB: long head of the biceps
MCI: minced cartilage implantation
MOCART: Magnetic Resonance Observation of Cartilage Repair Tissue
PRP: platelet‐rich plasma
ROM: range of motion
SD: standard deviation
SSV: Simple Shoulder Value
VAS: Visual Analogue Scale

## INTRODUCTION

For young patients with focal cartilage defects of the glenohumeral joint, several regenerative surgical techniques exist including microfracture [9, 16], osteochondral autograft transfer [21] and autologous chondrocyte implantation (ACI) [2, 3]. However, each of these techniques holds individual limitations, including either low quality of repair tissue, donor site morbidity or a two‐step surgical procedure [19].

Minced cartilage implantation (MCI), first reported in the early 1980s [1], is a technique for treatment of focal cartilage defects that has gained renewed interest due to a number of attributes, including being a single‐step procedure, offering strong biologic potential and relatively high cost‐effectiveness [20]. The technique entails harvesting viable autologous cartilage that is minced into small fragments and reimplanted. Autologous thrombin and platelet‐rich plasma (PRP) [15], fibrin glue [4] or a membrane [5] are additionally used for fragment fixation. Although promising short‐ to mid‐term clinical results of MCI have been reported for the knee joint [18, 22], to the best of our knowledge, results in the glenohumeral joint are limited to case reports [7, 12].

The purpose of the present study was to evaluate clinical and radiological outcomes after arthroscopic autologous MCI for focal cartilage defects of the glenohumeral joint. It was hypothesized that MCI would result in reliable clinical outcomes and sufficient defect coverage.

## MATERIALS AND METHODS

Patients who underwent arthroscopic autologous MCI for focal, Grade IV cartilage defects of the glenohumeral joint between October 2021 and March 2023, and had a minimum follow‐up of 2 years, were retrospectively analysed. The present study was approved by the institutional review board of the Technical University of Munich (IRB No. 2024‐477‐S‐CB) prior to commencement.

The indication for autologous MCI was restricted to contained, grade IV cartilage defects of the glenoid concavity or humeral head, defect size ≥1 cm^2^, intact subchondral plate and patient age <40 years. Patients undergoing concomitant surgical procedures were also included. Patients with osteoarthritis or kissing lesions with combined cartilage defects of the glenoid and humeral head were excluded. All patients underwent preoperative magnetic resonance imaging (MRI) of the affected shoulder to assess the grade, size and location of the cartilage defect as well as the integrity of the subchondral plate.

### Surgical technique

**Figure 1:**
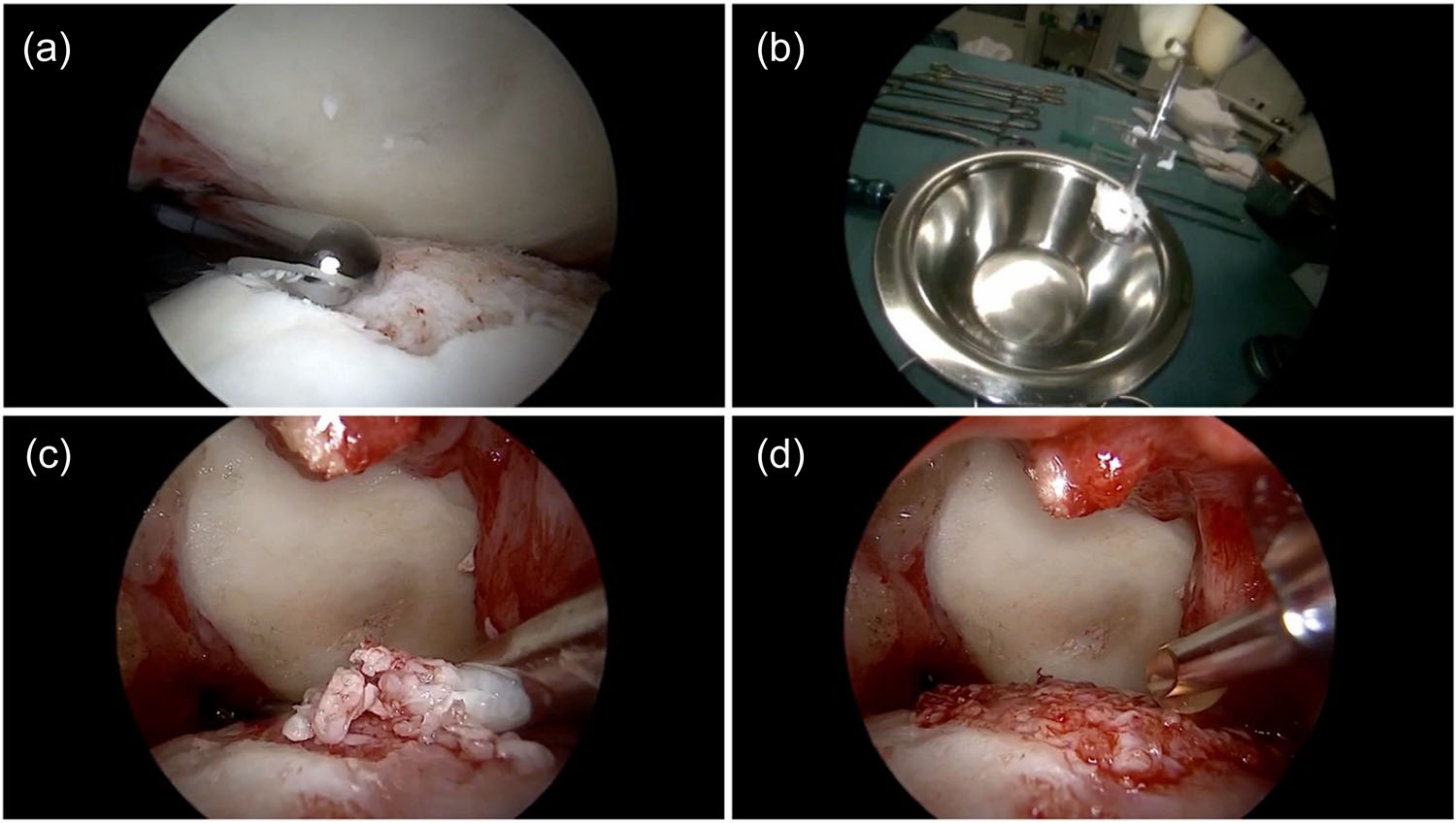
Healthy cartilage is harvested from the defect rim at the humeral head (Patient ID 8) and minced using a shaver system with a 3.0 mm blade (a). The cartilage fragments collected in the tissue collector demonstrate the recommended paste‐like appearance (b). The defect is covered with the mixture of cartilage fragments and ACP (c) and sealed with autologous thrombin (d). ACP, autologous conditioned plasma.

Patients were placed in the beach chair position. Diagnostic arthroscopy was performed to measure the cartilage defect and to identify any concomitant lesions, which were additionally treated. The cartilage defect was debrided, and a stable cartilage rim was obtained. The calcified layer was removed with the subchondral plate left intact. Cartilage fragments were harvested from the defect rim using a shaver system (Sabre, shaver blade, 3.0 mm; Arthrex GmbH) connected to a tissue collector (GraftNet™ autologous tissue collector; Arthrex GmbH) (Figure 1a,b).

For preparation of autologous conditioned plasma (ACP), 15 mL of venous blood was drawn using three double syringes (ACP® double syringe; Arthrex GmbH) and centrifuged at 1500 rpm for 5 min. The cartilage fragments were mixed with ACP in a ratio of 3:1. The fragment paste was then transferred into the application cannula.

The arthroscopic fluid was drained from the joint, and the lesion was dried with a swab. Using the application cannula, the fragment paste was applied into the defect to reach to about 80% of the defect's height (Figure 1c).

To produce the autologous thrombin solution, a total 9 mL of ACP was poured into the Thrombinator^TM^ system (Arthrex GmbH). The fragment paste was covered with the prepared thrombin serum (Figure 1d). For the final seal, ACP and thrombin in the ratio 1:1 was applied to the lesion. After 2 min, the arthroscopic instruments were removed.

### Postoperative rehabilitation

The operated shoulder was immobilized in an arm sling (medi GmbH & Co. KG) for 2 weeks. After 24 h, active mobilization exercises with pain level‐restricted range of motion (ROM) were allowed. Weight‐bearing exercises were initiated at 6 weeks. The rehabilitation protocol was adjusted in patients undergoing concomitant procedures.

### Clinical outcomes

Postoperative clinical outcome measures included the Constant–Murley (CM) score, the American Shoulder and Elbow Surgeons (ASES) score, the Simple Shoulder Value (SSV), the Disability of the Arm, Shoulder and Hand (DASH) score as well as the Visual Analogue Scale (VAS) for pain at rest and during exercise, which were collected at final follow‐up. Further, active and passive ROM of the operated and contralateral shoulder were evaluated. Shoulder strength for flexion, abduction, external rotation and internal rotation was measured relative to the contralateral side using an isometric dynamometer (ISOBEX; Cursor AG). Strength was obtained through three attempts each on the affected and contralateral sides; the mean value for each side was calculated, and subsequently the value for the affected side was extrapolated as a percentage relative to the contralateral side.

Postoperative shoulder‐dependent athletic ability was assessed using the Athletic Shoulder Outcome Scoring System (ASOSS). The ASOSS comprises the recording of subjective shoulder sport‐associated perception of pain, instability, muscular strength and endurance, intensity and proficiency level, as well as objective assessment of ROM. Each category is graded and compared to the before‐injury score, which is defined as 100% [25].

### Radiological outcomes

At final follow‐up, cartilage repair tissue morphology was analyzed on 3‐Tesla magnetic resonance imaging by three independent raters (one specialized musculoskeletal radiologist [K.W.], two specialized shoulder surgeons (B.S. and L.N.M.) using the Magnetic Resonance Observation of Cartilage Repair Tissue (MOCART) 2.0 score. The MOCART 2.0 score (range, 0–100 points) assesses the volume of cartilage defect filling (0–20 points), integration into adjacent cartilage (0–15 points), surface of the repair tissue (0–10 points), structure of the repair tissue (0–10 points), signal intensity of the repair tissue (0–15 points), bony defects or bony overgrowth (0–10 points) and subchondral changes (0–20 points) [23].

### Statistical analysis

Descriptive statistics including the median and interquartile range (IQR) for continuous variables as well as frequency and proportion for categorical variables were calculated to characterize the study population. Normality of continuous data was assessed via the Shapiro–Wilk test. The paired Wilcoxon test was used for the comparison of paired data for ROM and strength (affected and contralateral shoulder). To evaluate the agreement of the assessment of the MOCART 2.0 score, interclass correlation coefficients (ICC) were calculated. ICC < 0.5, 0.5–0.75, 0.75–0.9 and >0.9 were interpreted as poor, moderate, good and excellent reliability, respectively [14]. Results of inferential analyses are presented as 95% confidence intervals (CIs). A *p* value of less than 0.05 was considered statistically significant. All analyses were performed with SPSS software version 28.0.1.1 (IBM‐SPSS).

## RESULTS

All eight eligible patients (median age at surgery, 25.5 ± 5.5 years; six male and two female; follow‐up rate 100%) were included in the study, with a median follow‐up of 2.5 years (IQR 0.4; range, 2.0–2.8 years). The focal cartilage defect was located at the glenoid concavity in six patients and humeral head in two patients, with a median size of 2.4 cm^2^ (IQR 1.4; range, 1.0–4.0 cm^2^). In three patients (37.5%; ID 2, 3, 8), the dominant side was affected, while in five patients (62.5%; ID 1,4–7) the non‐dominant side was affected. Regarding type of sport, three patients participated in soccer (ID 1, 3, 4), three in fitness training (ID 5, 7, 8), one in gymnastics (ID 2) and one in climbing (ID 6).

Regarding the history of injury, three instability cases (ID 1, 3, 7) were included, in which the cartilage defect was caused by the traumatic event of shoulder dislocation. In these cases, MCI was performed concomitant with labral repair. Two patients (ID 4 and 5) had prior labral repair performed at an external institution due to a shoulder dislocation. During the index surgery, cartilage defects at the humeral head (ID 4) and glenoid (ID 5) were noticed, with subsequent referral of these patients to our institution for MCI in a second procedure. One patient (ID 6) had a traumatic cartilage defect at the glenoid due to a fall on the shoulder during climbing. The two patients with atraumatic cartilage defects at the glenoid (ID 2) and humeral head (ID 8) could not recall any traumatic event associated with the onset of symptoms. These patients reported gradual onset of symptoms during gymnastics (ID 2) or fitness training (ID 8). The surgical information of each included patient is summarized in Table 1.

**Table 1:** Surgical information of each patient.

ID	Age at surgery (years)	Sex	Aetiology	Prior surgeries	Localization of the cartilage defect	Defect size (cm^2^)	Concomitant procedures
**1**	31	Male	Traumatic		Glenoid	3.75	Anterior labral repair
2	20	Male	Atraumatic		Glenoid	1.00	None
3	28	Male	Traumatic		Glenoid	2.25	Anterior labral repair + LHB tenodesis
4	24	Male	Traumatic	Labral repair	Humeral head	2.00	None
5	26	Male	Traumatic	Labral repair	Glenoid	3.00	None
6	21	Male	Traumatic		Glenoid	1.00	None
7	25	Female	Traumatic		Glenoid	2.50	Posterior labral repair
8	39	Female	Atraumatic		Humeral head	4.00	LHB tenodesis

Abbreviations: ID, patient identification; LHB, long head of the biceps.

### Clinical outcomes

At final follow‐up, patients showed a median CM score of 85.0 (IQR 13.0), CM score relative to the contralateral side of 95.5 (IQR 7.1), ASES score of 93.5 (IQR 10.5), SSV of 90.0 (IQR 13.8) and DASH score of 4.6 (IQR 6.7). The median DASH sport and music subcategory was 9.4 (IQR 15.6), while the median DASH work subcategory was 0.0 (IQR 1.6). The median VAS for pain was 0.0 (IQR 0.0) at rest and 2.0 (IQR 1.3) during exercise (Table 2).

**Table 2:** Functional scores for each patient.

ID	FU (mo)	CM	CMr (%)	ASES	DASH	DASHsm	DASHw	SSV	VASr	VASe	ASOSS
1	31	77	95	88	6	6	0	90	0	2	82
2	33	96	96	92	3	13	0	95	1	2	86
3	31	82	97	100	2	0	0	90	0	1	82
4	29	78	88	95	7	0	6	70	0	2	94
5	26	88	91	88	16	25	0	85	0	2	86
6	24	93	101	100	3	13	0	95	0	0	98
7	32	57	64	58	37	94	56	50	0	5	54
8	27	90	99	98	1	0	0	96	0	0	88

Abbreviations: ASES, American Shoulder and Elbow Surgeons score; ASOSS, Athletic Shoulder Outcome Scoring System; CM, Constant–Murley score; CMr, Constant–Murley score relative to contralateral side; DASH, Disability of the Arm, Shoulder and Hand score; DASHsm, DASH sport and music subcategory; DASHw, DASH work subcategory; FU, follow‐up in months; ID, patient identification; SSV, Simple Shoulder Value; VASe, Visual Analogue Scale for pain during exercise; VASr, Visual Analogue Scale for pain at rest.

Patients demonstrated no significant differences for active and passive flexion, abduction, external rotation and internal rotation compared to the contralateral side (*p* > 0.05, respectively). Further, there were no significant differences in strength during flexion, abduction, external rotation and internal rotation relative to the contralateral side (*p* > 0.05, respectively).

With a median ASOSS of 86.0 (IQR 7.5), patients showed good shoulder‐dependent athletic ability.

### Radiological outcomes

**Figure 2:**
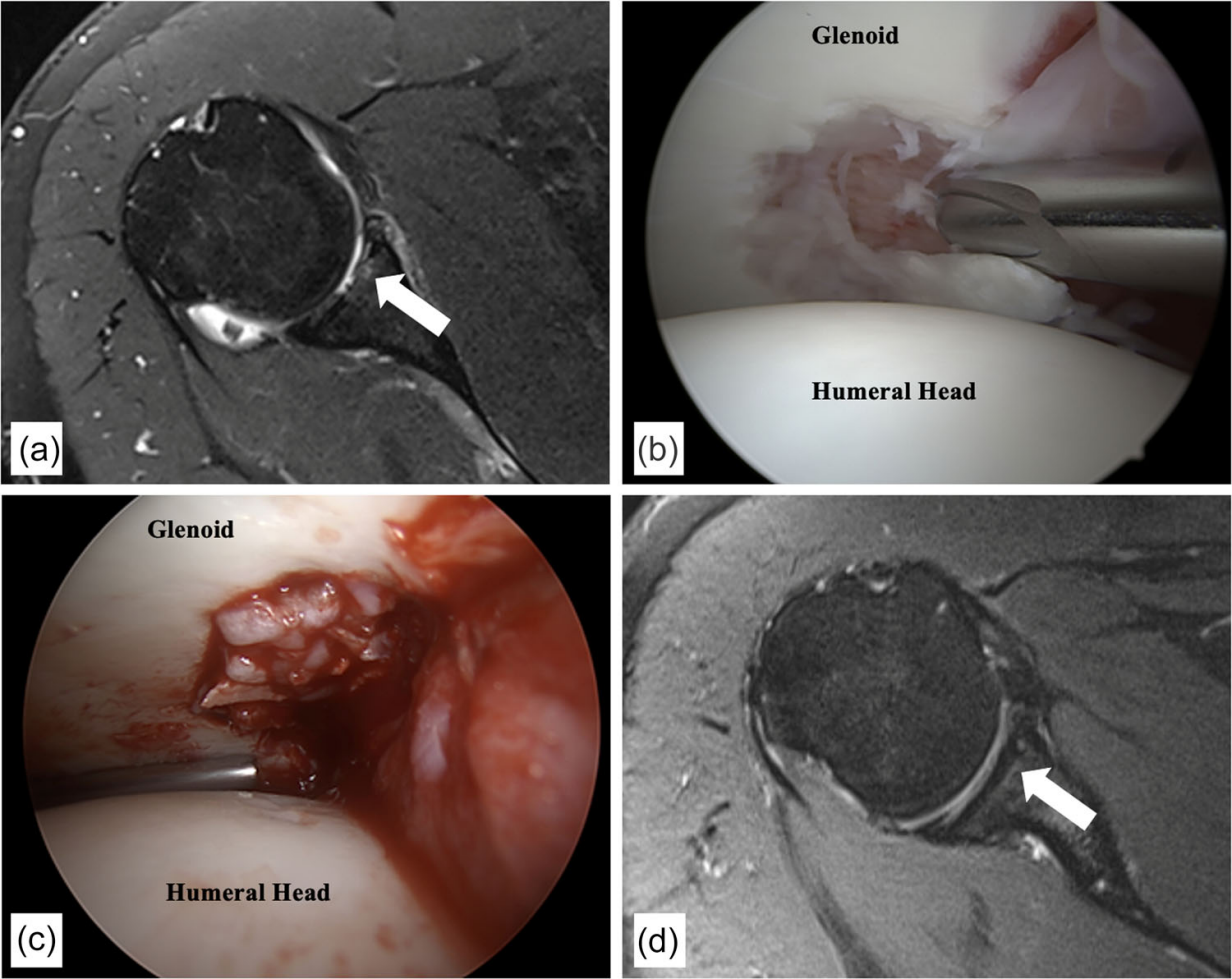
Preoperative MRI scan in the axial plane of Patient ID 3 showing a cartilage defect at the anterior glenoid surface (a). The MRI, 31 months after MCI, shows successful defect coverage (d). Intraoperative images of the same patient demonstrating the cartilage defect prior to debridement (b) and after MCI (c). MCI, minced cartilage implantation; MRI, magnetic resonance imaging.

**Figure 3:**
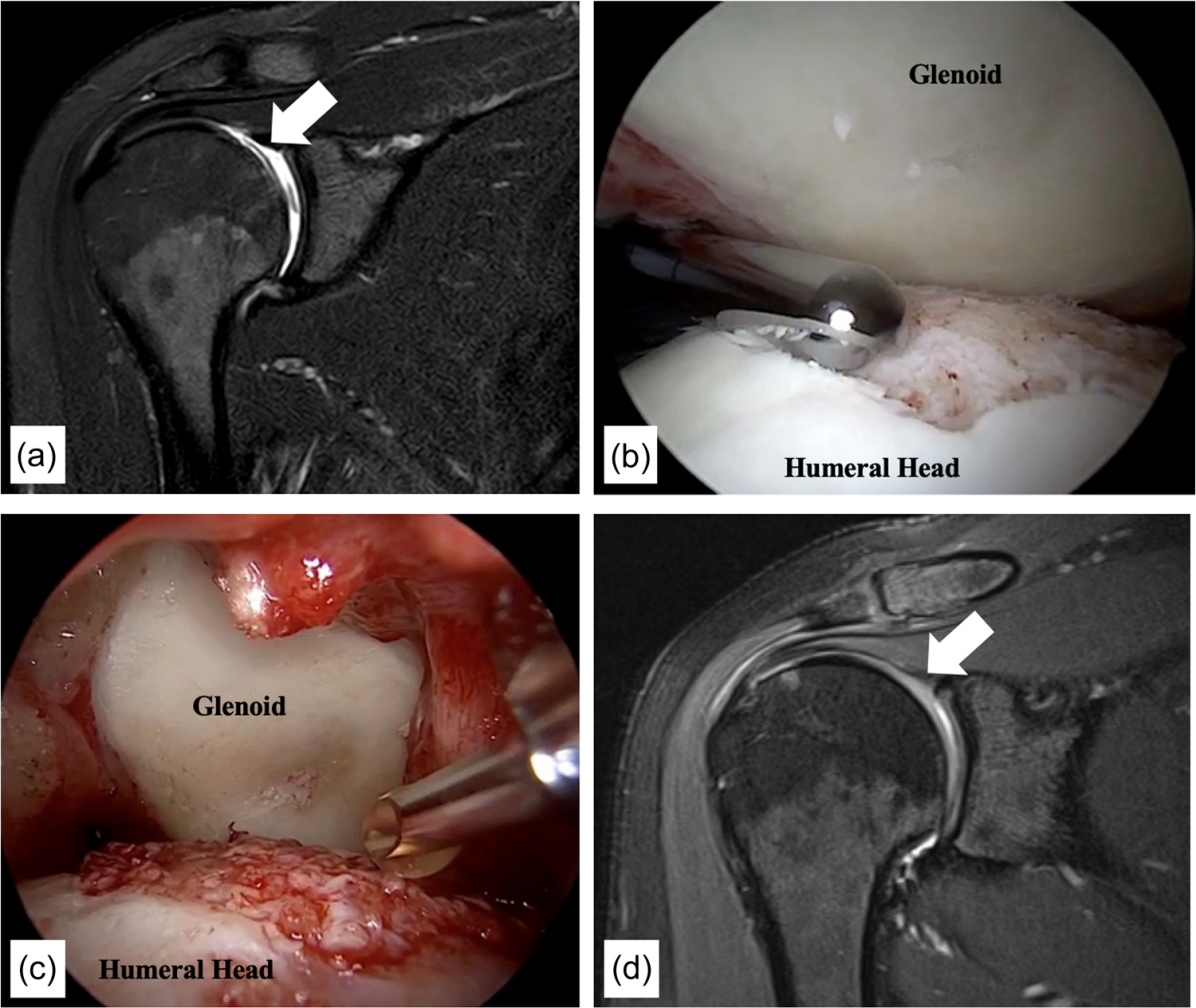
Preoperative MRI scan in the coronal plane of Patient ID 8 showing a cartilage defect at the humeral head (a). Intraoperative images of the same patient demonstrate the cartilage defect prior to debridement (b) and after MCI (c). The MRI after 27 months shows successful defect coverage (d). MCI, minced cartilage implantation; MRI, magnetic resonance imaging.

The median MOCART 2.0 score was 74.2 (IQR 13.8) with an excellent interrater reliability (ICC 0.891; 95% CI 0.576–0.983) (Table 3, Figures 2 and 3). The median values for the subcategories of the MOCART 2.0 score were 17.5 (IQR 3.8) for volume fill of cartilage defect, 11.7 (IQR 5.0) for integration into adjacent cartilage, 5.0 (IQR 1.7) for surface of the repair tissue, 0.0 (IQR 5.0) for structure of the repair tissue, 10.0 (IQR 5.0) for signal intensity of repair tissue, 10.0 (IQR 0.0) for bony defect/overgrowth and 20.0 (IQR 4.1) for subchondral changes.

**Table 3:** Radiological outcomes based on the MOCART 2.0 score for each patient.

ID	Volume fill (20)	Integration (15)	Surface of repair tissue (10)	Structure of repair tissue (10)	Signal intensity of repair tissue (15)	Bony defect (10)	Subchondral changes (20)	Total (100)
1	16.7	8.3	5	3.3	10	10	16.3	70.0
2	18.3	11.7	6.7	0	6.7	10	20	73.3
3	20	15	6.7	0	10	10	20	81.7
4	16.7	10	5	0	10	5	15	61.7
5	5	11.7	3.3	10	15	10	15	70.0
6	20	15	10	0	15	10	20	90.0
7	15	15	5	0	10	10	20	75.0
8	20	10	5	10	15	10	20	90.0

*Note*: Values are reported as mean between three raters. The maximum score for each subcategory is stated in brackets

Abbreviations: ID, patient identification; MOCART, Magnetic Resonance Observation of Cartilage Repair Tissue.

### Complications and revisions

No complications related to MCI were observed, and none of the patients underwent revision surgery. However, one patient (ID 7) showed severe clinically symptomatic long head of the biceps (LHB) tendinitis at final follow‐up, as demonstrated in the significantly worse functional outcome scores compared to the other patients. This was a patient with an initial Moroder Type A2 posterior shoulder instability, who had progressed to a static Moroder Type C2 situation over time despite posterior labral repair and subsequently developed concomitant LHB tendinitis.

## DISCUSSION

The most important finding of the present study was that arthroscopic autologous MCI is a reliable treatment option for focal, full‐thickness cartilage defects of the glenohumeral joint in young and active patients. At a minimum 2‐year follow‐up, patients showed successful coverage of the cartilage defect along with good to excellent functional outcomes and shoulder‐dependent athletic ability in patients with or without concomitant procedures.

Recently, Schneider et al. reported functional and radiological outcomes of 62 patients after arthroscopic autologous MCI for focal cartilage defects of the knee joint using an equivalent, standardized technique compared to the present study [22]. At a mean follow‐up of 2 years, the authors found satisfying functional results and a mean MOCART 2.0 score of 62.9 ± 9.9 among 20 available patients [22]. For the MOCART 2.0 subcategories, there was good defect filling (14.5 ± 3.9) and integration of the repair tissue (11.6 ± 3.7), while surface (5.4 ± 2.9) and structure (1.4 ± 3.4) showed only fair to poor results [22].

This is consistent with our radiological observation, resulting in a median MOCART 2.0 score of 74.2, including substantial defect coverage, but poorer results for the subcategories surface (5.0) and structure (0.0). These outcomes highlight the potential capability of autologous MCI in restoration of glenohumeral cartilage defects at a 2‐year follow‐up. However, questions regarding the quality of the repair tissue and the potential to delay progression of osteoarthritis require further research.

Although focal cartilage defects of the glenohumeral joint are markedly less common compared to the knee joint, re‐establishing the articular surface is of importance to prevent progression of degeneration, especially in young patients [8, 24]. Regenerative surgical techniques for glenohumeral cartilage defects primarily include microfracture, osteochondral autograft transfer and ACI [6, 24]. However, each of these techniques has been shown to hold some inherent limitations [6, 24].

While substantial improvement in pain and shoulder function has been reported after microfracture, this technique should be limited to small diameter cartilage defects, as in patients with larger lesions significantly worse outcomes have been reported [9, 16, 26]. Further, outcome deterioration may be expected with time due to the low quality of the regenerative fibrocartilage [11, 24]. Microfracture was also not found to prevent radiological progression of osteoarthritis in the long‐term [11, 26]. When performing an osteochondral autograft transfer from the knee to the shoulder, donor site morbidity is perhaps the most important risk factor. Clinical evidence of this technique is limited to a small case series of eight patients, demonstrating sufficient functional and radiographic outcomes; however, a significant progression of osteoarthritic changes was observed in the longer term [13, 21].

ACI can be regarded as a highly effective tissue engineering procedure, with lesion diameter and depth not precluding its use. Previous investigations of ACI at the glenohumeral joint comprise two case series with short‐ to mid‐term follow‐up [2, 3]. At a mean 41‐month follow‐up, Buchmann et al. reported satisfactory clinical outcomes, including VAS for pain of 0.3, CM score of 83.3, and ASES score of 95.3, after ACI using a Type I/III collagen‐based membrane for three humeral‐sided and one glenoid‐sided cartilage defect [3]. Further, the MOCART score was indicative of satisfactory defect coverage with signs of fibrocartilaginous repair tissue [3]. Boehm et al. observed sufficient clinical results and successful defect coverage with only minor radiologic degenerative changes at 32 months in seven patients following ACI using three‐dimensional spheroids of human autologous matrix‐associated chondrocytes [2]. However, major limitations of ACI include that two surgeries are required and the cost intensiveness [2, 3].

The potential alternative to ACI may be autologous MCI, representing a single‐step procedure, further facilitated by a substantially simplified approval process. However, clinical results after autologous MCI in the glenohumeral joint are currently limited to descriptions of surgical techniques [15, 17] and case reports comprising a total of two patients [7, 12]. Karkosch et al. reported a significant improvement in CM (50–98), ASES (40–98.3) and University of California Los Angeles Shoulder (UCLAS) (16–35) scores along with pain relief (Numeric Rating Scale [NRS] 6–0) in one patient at 6‐month follow‐up [12]. The postoperative MRI revealed a thin cartilage layer in the former defect area with sufficient integration to the surrounding tissue [12]. In another case report, the patient was pain‐free with an SSV of 95% and a CM score of 95 at 1 year after combined labral repair and MCI for treatment of a glenolabral articular disruption (GLAD) lesion, while MRI showed good coverage of the cartilage defect [7].

Similarly, the present study observed a median CM score of 85.0, CM score relative to the contralateral side of 95.5, ASES score of 93.5, SSV of 90.0 and DASH score of 4.6, along with no significant differences for active and passive ROM as well as strength compared to the contralateral side. Further, the MRI investigation showed a mean MOCART 2.0 score of 74.2, indicating substantial healing of the cartilage defect. It is important to note that in the present surgical technique, ACP was additionally applied, which has been shown to enhance proteoglycan production of chondrogenic spheroids in vitro, which may be beneficial compared to MCI without augmentation [10]. However, the currently available evidence for functional and radiographic outcomes after MCI for the glenohumeral joint clearly highlights the need for outcome studies with larger, homogeneous sample sizes and longer follow‐up.

## LIMITATIONS

There are limitations to the study. First, the patient population was small; however, this reflects the rare indication for treatment of focal cartilage defects in the glenohumeral joint. Second, the study inherits the associated biases of a retrospective design. As no baseline values of functional scores were available, the true improvement of each patient from pre‐ to postoperatively remains unknown. Third, a focal cartilage defect of the humeral head was treated with arthroscopic autologous MCI only in two patients, limiting the conclusion about the chances of success of the therapy at this specific location. Although the used clinical outcome scores are common for assessment of shoulder function, they are not specifically validated for focal cartilage defects in the glenohumeral joint. Finally, the subject population was heterogeneous in terms of previous surgeries and concomitant procedures, which may affect functional outcomes more than the performed MCI procedure itself.

## CONCLUSION

MCI for treatment of focal cartilage defects of the glenohumeral joint showed a radiologically successful defect coverage along with good to excellent clinical outcomes in patients with or without concomitant procedures at a minimum 2‐year follow‐up.

## AUTHOR CONTRIBUTIONS

*Conceptualization*: Lukas Nawid Muench and Bastian Scheiderer. *Methodology*: Lukas Nawid Muench, Bastian Scheiderer and Klaus Woertler. *Formal analysis and investigation*: Lukas Nawid Muench and Thilo Demmer. *Writing—original draft preparation*: Lukas Nawid Muench, Bastian Scheiderer and Thilo Demmer. *Writing—review and editing*: Sebastian Siebenlist, Lucca Lacheta and Klaus Woertler. *Funding acquisition*: No funding. *Resources*: Sebastian Siebenlist, Bastian Scheiderer and Klaus Woertler. *Supervision*: Lucca Lacheta, Bastian Scheiderer, Sebastian Siebenlist and Klaus Woertler.

## FUNDING INFORMATION

The authors have no funding to report.

## CONFLICT OF INTEREST STATEMENT

Bastian Scheiderer is a consultant for Arthrex GmbH. Sebastian Siebenlist is a consultant for Arthrex GmbH, KLS Martin Group and medi GmbH & Co. KG. The remaining authors declare no conflicts of interest.

## ETHICS STATEMENT

This retrospective chart review study involving human participants was in accordance with the ethical standards of the institutional and national research committee and with the 1964 Helsinki Declaration and its later amendments or comparable ethical standards. The Human Investigation Committee (IRB) of the Technical University of Munich approved this study (IRB No. 2024‐477‐S‐CB). Informed consent was obtained from all individual participants included in the study.

## Data Availability

The DAS confirms the presence or absence of data.
